# Dietary fructose enhances the incidence of precancerous hepatocytes induced by administration of diethylnitrosamine in rat

**DOI:** 10.1186/2047-783X-18-54

**Published:** 2013-12-09

**Authors:** Ryo Kumamoto, Hirofumi Uto, Kohei Oda, Rie Ibusuki, Shirou Tanoue, Shiho Arima, Seiichi Mawatari, Kotaro Kumagai, Masatsugu Numata, Tsutomu Tamai, Akihiro Moriuchi, Hiroshi Fujita, Makoto Oketani, Akio Ido, Hirohito Tsubouchi

**Affiliations:** 1 Digestive and Lifestyle Diseases, Department of Human and Environmental Sciences, Kagoshima University Graduate School of Medical and Dental Sciences, 8-35-1 Sakuragaoka, Kagoshima, Kagoshima 890-8544, Japan; 2 Department of HGF Tissue Repair and Regenerative Medicine, Kagoshima University Graduate School of Medical and Dental Sciences, 8-35-1 Sakuragaoka, Kagoshima, Kagoshima 890-8544, Japan

**Keywords:** Diethylnitrosamine, Fructose, Hepatocarcinogenesis, Nonalcoholic steatohepatitis, Placental form of glutathione-S-transferase

## Abstract

**Background:**

Nonalcoholic fatty liver disease (NAFLD) is a risk for hepatocellular carcinoma (HCC), but the association between a high-fructose diet and HCC is not fully understood. In this study, we investigated whether a high-fructose diet affects hepatocarcinogenesis induced by administration of diethylnitrosamine (DEN).

**Methods:**

Seven-week-old male Sprague–Dawley rats were fed standard chow (controls), a high-fat diet (54% fat), or a high-fructose diet (66% fructose) for 8 weeks. All rats were given DEN at 50 μg/L in drinking water during the same period. Precancerous hepatocytes were detected by immunostaining of the placental form of glutathione-S-transferase (GST-P). The number of GST-P-positive hepatocytes was assessed in liver specimens.

**Results:**

Serum levels of total cholesterol were similar among the three groups, but serum triglyceride, fasting blood glucose, and insulin levels were higher in the high-fructose group compared to the high-fat group. In contrast, hepatic steatosis was more severe in the high-fat group compared with the high-fructose and control groups, but the incidence of GST-P-positive specimens was significantly higher in the high-fructose group compared to the other two groups. The average number of GST-P-positive hepatocytes in GST-P positive specimens in the high-fructose group was also higher than those in the other two groups. This high prevalence of GST-P-positive hepatocytes was accompanied by higher levels of 8-hydroxydeoxyguanosine in serum and liver tissue.

**Conclusions:**

These results indicate that dietary fructose, rather than dietary fat, increases the incidence of precancerous hepatocytes induced by administration of DEN via insulin resistance and oxidative stress in rat. Thus, excessive fructose intake may be a potential risk factor for hepatocarcinogenesis.

## Background

Fatty liver is roughly divided into alcoholic and nonalcoholic fatty liver diseases. Nonalcoholic fatty liver disease (NAFLD) includes nonalcoholic fatty liver without hepatocellular injury and fibrosis, and nonalcoholic steatohepatitis (NASH) accompanied by liver inflammation and hepatocyte injury with a risk of hepatic cirrhosis and hepatocellular carcinoma (HCC) [1,2]. In a study of 420 subjects diagnosed with NAFLD and followed for a mean period of 7.6 years, Adams et al. reported that 21 patients were diagnosed with liver cirrhosis at final follow-up, indicating that about 1% of NAFLD cases progressed to liver cirrhosis yearly [3]. Progression from NASH-associated liver cirrhosis to HCC within 5 years occurs at a higher rate of 11.3%, although this is still lower than the 30.5% rate of progression of hepatitis C virus (HCV)-associated liver cirrhosis [4]. High frequencies of HCC (50%) and hepatic insufficiency (33%) have also been reported as causes of death from NASH-associated liver cirrhosis [5]. Thus, compared to HCV-associated chronic liver disease, the frequency of progression of NAFLD/NASH to liver cirrhosis or subsequent complication of HCC is low. However, the prevalence of NAFLD in thorough medical checkups has been estimated to be about 20% [6], showing that the number of NAFLD patients is high compared to those with HCV-associated chronic liver disease. Therefore, NAFLD/NASH progresses to HCC in a relatively large number of patients, and there are concerns that this number may increase further in the future.

Obesity- or abnormal glucose tolerance-associated insulin resistance induces hepatic steatosis (‘first hit’); subsequently, NASH develops following a complex process due to ‘second hits’ such as oxidative stress, inflammatory cytokines, iron, and endotoxin [7]. Obesity and insulin resistance, which are the main pathogenic factors in NASH, have also been shown to be carcinogenic risk factors for HCC [8,9]. However, further investigation of the risk factors and molecular mechanisms underlying hepatocarcinogenesis is required.

Fructose is referred to as ‘fruit sugar’ because it is abundant in fruits and is considered to be the sweetest among all natural sugars. Sucrose (the main component of sugar) is hydrolyzed to glucose and fructose. Common refreshing beverages contain a high level of fructose and excess consumption of fructose is associated with exacerbation of obesity and insulin resistance [10-12], which are risk factors for HCC. Fructose may also promote liver fibrosis in fatty liver disease [13]. These findings suggest that excess fructose consumption may be associated with development of HCC from NASH, but this association has not been fully investigated.

We previously suggested that the pathophysiology of fatty liver disease may be determined by its etiology, rather than by its severity [14]. The severity of hepatic steatosis induced by a high-fructose diet is milder than that induced by a high-fat diet. However, liver regeneration after hepatectomy was significantly delayed with the high-fructose diet compared to the high-fat diet in an animal model [14]. In this study, we investigated whether the appearance of glutathione S-transferase placental type (GST-P)-positive hepatocytes, which are thought to be precancerous lesions of HCC [15], differed according to the etiology of fatty liver disease.

## Methods

### Animals and diets

Seven-week-old male Sprague–Dawley rats (Kyudo, Kumamoto, Japan) were fed a high-fat diet (54% of the total energy [4.96 kcal/g] comprised of fat, 8% from fructose), a high-fructose diet (total energy: 3.97 kcal/g, fructose: 66%, fat: 11%), or a control diet (total energy: 3.81 kcal/g, fat: 12%, fructose: 10%) (all obtained from Nosan Corporation, Kanagawa, Japan). Diethylnitrosamine (DEN) was administered in drinking water at a concentration of 50 μg/L [16] during the dietary period. The maximal daily food intake was limited to 30 g. After 8 weeks, the animals (8 in each group) were sacrificed under anesthesia by intraperitoneal administration of pentobarbital and serum and liver tissue were collected. The liver and serum samples were frozen at −80°C until measurement. All animal experiments were approved by the Institutional Animal Care and Use Committee of Kagoshima University.

### Evaluation of serum markers

Blood samples were collected from each rat via aortic punctures after a fast of 12 h. Serum levels of alanine aminotransferase (ALT), total cholesterol, and triglycerides were tested using an automatic analyzer (7180 Clinical Analyzer; Hitachi High-Technologies Corporation, Tokyo, Japan). Fasting blood glucose was measured using a glucometer (Freestyle Freedom; Nipro Co., Ltd., Osaka, Japan). Serum immunoreactive insulin levels were determined by ELISA (Morinaga Institute of Biological Science, Kanagawa, Japan).

### Assessment of hepatic mRNA levels

Total RNA was extracted using Isogen (Nippon Gene, Tokyo, Japan). The relative levels of specific mRNAs in liver were assessed by real-time quantitative polymerase chain reaction (PCR) using Syber Premix Ex Taq (TaKaRa Bio Inc., Shiga, Japan). Expression levels of target genes were calculated relative to the level of β-actin. All procedures were performed according to manufacturers’ instructions. The PCR primers were obtained from TaKaRa Bio Inc. Gene expression was analyzed using the following pairs of primers: tumor necrosis factor (TNF)-α (forward, TCA GTT CCA TGG CCC AGA C; reverse, GTT GTC TTT GAG ATC CAT GCC ATT); interferon (IFN)-γ (forward, AGG CCA TCA GCA ACA ACA TAA GTG; reverse, GAC AGC TTT GTG CTG GAT CTG TG); hemeoxygenase (HO)-1 (forward, AGG TGC ACA TCC GTG CAG AG; reverse, TCC AGG GCC GTA TAG ATA TGG TAC A); and β-actin (forward, GGA GAT TAC TGC CCT GGC TCC TA; reverse, GAC TCA TCG TAC TCC TGC TTG CTG).

### Oxidative stress marker

Serum and liver tissue levels of 8-hydroxydeoxyguanosine (8-OHdG), a marker of oxidative stress-induced DNA damage, were measured using an 8-OHdG Check ELISA kit (Japan Institute for the Control of Aging, Nikken SEIL Co., Ltd., Shizuoka, Japan).

### Histological examination

Tissue samples were fixed overnight in 10% phosphate-buffered formaldehyde, embedded in paraffin, and stained with hematoxylin and eosin or Azan. Oil red O staining was performed to evaluate accumulation of fat droplets in hepatocytes in frozen liver sections. The ratio of the oil red O-stained area to the total area was measured on a video display (100× magnification) in a blinded manner using a QuickGrain digital image analyzer (Inotech, Hiroshima, Japan).

Immunohistochemical analyses of GST-P (Medical & Biological Laboratories Co., Ltd., Nagoya, Japan) were performed using paraffin-embedded sections [17]. Samples were incubated with goat anti-rabbit IgG (Nichirei Biosciences Inc., Tokyo, Japan) to detect bound antibodies. The number of GST-P-positive hepatocytes in liver specimens from each rat was counted in 15 random fields at 100× magnification [17]. The incidence refers to the percentage of rats bearing GST-P-positive hepatocytes in each group, while the multiplicity represents the average number of GST-P-positive hepatocytes in positive specimens.

### Statistical analysis

Data are shown as the mean ± standard deviation (SD). Statistical comparison among groups was performed using Kruskal-Warris or χ^2^ test, and group means were considered to be significantly different at *P* <0.05 as determined by Games-Howell test.

## Results

### General appearance and serum parameters

**Figure 1:**
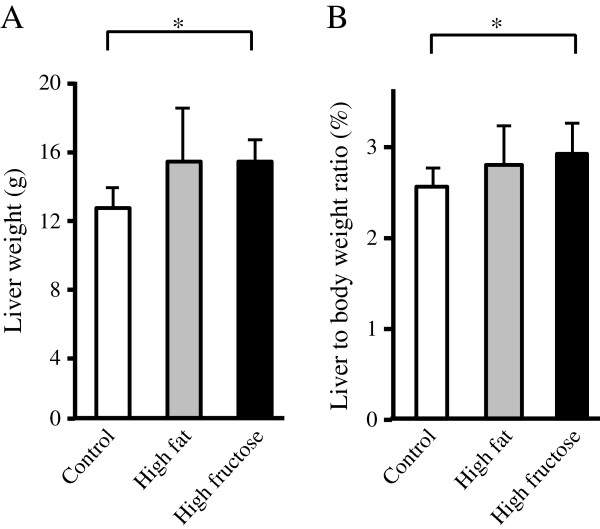
**Liver weight and liver to body weight ratio.** The liver weight **(A)** and the ratio of liver weight to body weight **(B)** were higher in the high-fructose group compared to the control group, and tended to be higher in the high-fat group compared to the control group. Values are mean ± standard deviation of 8 rats. *P <0.05*.*

Body weight in the high-fat group (body weight [mean ± SD] =529 ± 64 g) was higher than that in the high-fructose (506 ± 32 g) or control groups (489 ± 25 g) after the 8-week experimental period with oral intake of DEN, but there was no significant difference since the maximal food intake was limited to 30 g/day/animal in all groups. In contrast, liver weight and liver to body weight ratio were significantly or tended to be higher in the high-fructose or high-fat groups compared to the control group after 8 weeks, respectively (Figure 1).

Serum levels of ALT were significantly different among the three groups and were higher in the high-fat and high-fructose groups compared to the control group (Table 1). Serum total cholesterol levels were similar among the three groups, but serum triglyceride was significantly lower in the high-fat group compared to the high-fructose and control groups. Fasting blood glucose levels were higher in the high-fructose group compared to the high-fat group. Serum insulin levels were also higher in the high-fructose group compared to the high-fat group (Table 1).

**Table 1:** Serum biochemical markers after 8 weeks of diet intake

	**Control**	**High fat**	**High fructose**	***P *** value*
ALT (IU/L)	13.5 ± 5.2	26.1 ± 9.9	23.9 ± 10.0	0.01
Total cholesterol (mg/dL)	60.8 ± 7.7	57.6 ± 6.4	69.0 ± 15.7	0.37
Triglyceride (mg/dL)	104.3 ± 37.3	49.1 ± 13.2^#^	149.7 ± 54.6^##^	0.001
Fasting blood glucose (mg/dL)	133.4 ± 15.9	109.8 ± 31.3^#^	151.6 ± 14.2^##^	0.01
Insulin (ng/mL)	3.58 ± 1.89	3.23 ± 1.27	5.92 ± 1.80^##^	0.03

Data are shown as mean ± standard deviation. *ALT*, Alanine aminotransferase. *P values determined by Kruskal-Wallis test. ^#^*P* <0.05 vs. control group (Games-Howell test). ^##^*P* <0.05 vs. high-fat group (Games-Howell test).

### Hepatic steatosis and fibrosis

**Figure 2:**
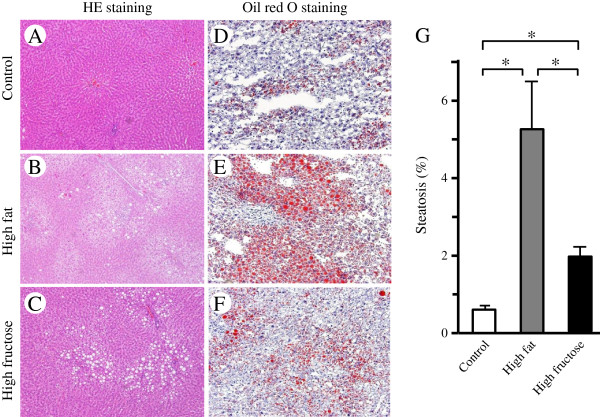
**Effects of a high-fructose diet with DEN intake on hepatic steatosis. (A-C)** Fat droplet deposition evaluated by hematoxylin-eosin staining was observed in the liver in rats fed a high-fat diet **(B)** and a high-fructose diet **(C)**, but not in those fed a control diet **(A)**. In addition, hepatic fibrosis was not observed in any of these groups. **(D-F)** Oil red O staining showed that fat deposition was more marked in the high-fat group **(E)** than in the high-fructose group **(F)**, and this difference was significant **(G)**. Values are shown as mean ± standard deviation of 8 rats. *P <0.05*.*

Histological evaluation by hematoxylin and eosin staining showed fat droplet deposition in hepatocytes in the high-fructose and high-fat groups (Figure 2B,C), but no apparent liver fibrosis in any group (Figure 2A-C). In addition, oil red O staining showed more marked fat deposition in the high-fat group (Figure 2E) than in the control or high-fructose groups (Figure 2D or F, respectively), and this difference was significant (Figure 2G).

### Precancerous hepatocytes induced by oral administration of DEN

**Figure 3:**
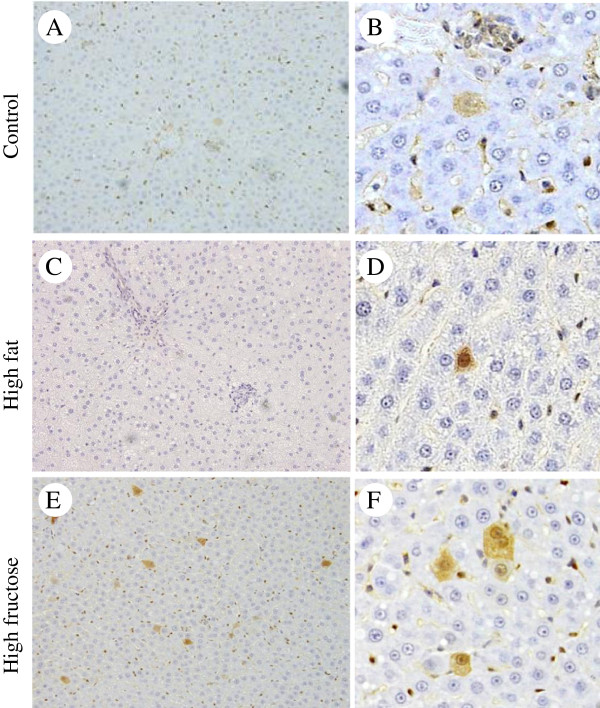
**Effects of a high-fructose diet and DEN intake on precancerous GST-P-positive hepatocytes. (A-F)** Immunohistochemical staining of GST-P in liver sections of rats given a control diet **(A,B)**, a high-fat diet **(C,D)**, or a high-fructose diet **(E,F)** with DEN for 8 weeks (**A**, **C** and **E**, magnification 100×; **B**, **D** and **F**, magnification 400×). The number of GST-P-positive hepatocytes seems to be greater in the high-fructose group than in the high-fat group.

Liver tissue was evaluated by GST-P staining because GST-P-positive cells are thought to be characteristic of precancerous lesions [15]. There were more GST-P-positive hepatocytes in the high-fructose group (Figure 3E,F) compared to the high-fat group (Figure 3C,D). One slide was randomly selected from each of the left, right, and middle lobes and evaluated for each rat in each group (3 slides × 8 animals = 24 slides/group). The ratio of slides containing GST-P-positive hepatocytes differed significantly among the three groups (*P* = 0.03), with the highest ratio (62.5%; 15/24) in the high-fructose group followed by the high-fat group (Table 2).

**Table 2:** Precancerous hepatocytes induced by oral administration of DEN with 8 weeks of diet

	**Control**	**High fat**	**High fructose**	***P *** value*
The number of slides containing GST-P-positive hepatocytes in 24 slides	6	9	15	0.03
The number of GST-P-positive hepatocytes in 360 fields	7	12	212	<0.001
The number of GST-P-positive hepatocytes in GST-P positive slides	1.17 ± 0.41	1.33 ± 0.71	14.1 ± 37.9	0.03

Data are shown as the number or mean ± standard deviation. *P values determined by χ^2^ test or Kruskal-Wallis test, as appropriate.

The slides from each of the left, right, and middle lobes were also observed at 100× magnification and 15 visual fields from each slide were randomly selected (3 slides × 15 visual fields/animal × 8 rats/group = 360 visual fields in each group). The number of GST-P-positive hepatocytes was highest in the high-fructose group (Table 2, *P* <0.001), whereas the numbers were small in the high-fat and control groups. The mean number of GST-P-positive hepatocytes in positive slides was 14.1 in the high-fructose group, which was greater than those in the high-fat and control groups (Table 2).

### Hepatic mRNA expression levels in the high-fructose and high-fat groups

**Figure 4:**
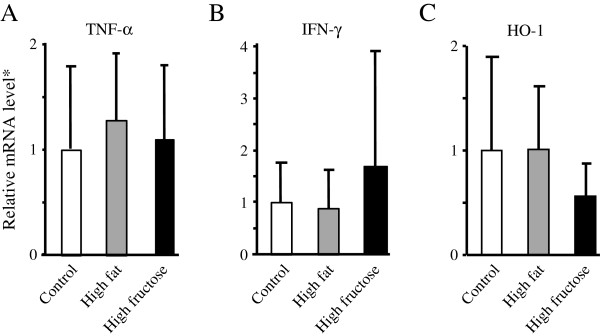
**Hepatic mRNA expression of tumor necrosis factor (TNF)-α, interferon (IFN)-γ and hemeoxygenase (HO)-1 assessed by reverse transcription-quantitative PCR after 8 weeks of dietary intake with diethylnitrosamine in drinking water. (A)** Hepatic expression TNF-α mRNA in rats fed a high-fructose diet was similar to that in rats fed a high-fat or control diet. **(B)** Hepatic expression of IFN-γ mRNA tended to be higher in rats fed a high-fructose diet compared to that in rats fed a high-fat or control diet. **(C)** Hepatic expression of HO-1 mRNA tended to be lower in rats fed a high-fructose diet compared to that in rats fed a high-fat or control diet. *mRNA level normalized using that of β-actin. Values are means ± standard deviation of 8 rats.

Hepatic expression levels of TNF-α mRNA were similar among the three groups (Figure 4A). In contrast, hepatic levels of IFN-γ mRNA were 1.9- and 1.7-fold higher in the high-fructose group compared to those in the high-fat and control groups, respectively (Figure 4B). Hepatic mRNA expression of HO-1 was lower in the high-fructose group compared to the high-fat and control groups (Figure 4C), but these differences did not reach statistical significance.

### Higher oxidative stress in rats with a high-fructose diet

**Figure 5:**
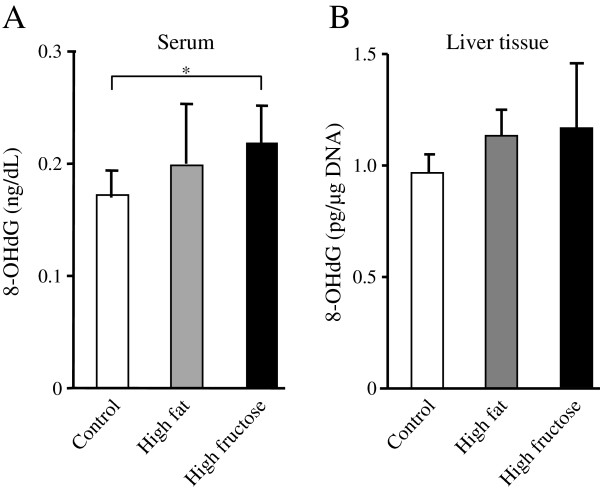
**Serum and liver tissue levels of 8-hydroxydeoxyguanosine (8-OHdG). (A)** The serum 8-OHdG level differed significantly among the three groups (*P* = 0.03), and this level tended to be or was significantly higher in the high-fructose group than in the high-fat or control groups, respectively. Values are means ± standard deviation of 8 rats. *P <0.05. **(B)** The 8-OHdG level in liver tissue tended to be higher in the high-fructose group (n = 8) compared to that in the high-fat (n = 7) or control groups (n = 8).

The serum 8-OHdG level differed significantly among the three groups (*P* = 0.03); this level was significantly higher in the high-fructose group and showed a tendency to be higher in the high-fat group compared to the control group (Figure 5A). The 8-OHdG level in liver tissue tended to be higher in the high-fructose group compared to the control group and the high-fat group (Figure 5B).

## Discussion

NAFLD/NASH and its accompanying pathophysiology, severe obesity, and glucose intolerance are important risk factors for HCC [8,9,18] and an increase in the number of patients with HCC with a background of metabolic syndrome is of major concern. Excessive intake of fat or fructose is also a potential risk factor for hepatocarcinogenesis [17,19]; however, the underlying mechanisms are not well understood. Further, it is unclear whether development of HCC and the mechanism of hepatocarcinogenesis differ due to differences in the etiology of fatty liver disease. In this study, we used high-fat and high-fructose diets to prepare abnormal lipid-induced and abnormal glucose metabolism-induced hepatic steatosis, respectively. Rats were exposed to DEN in drinking water throughout the study to induce formation of GST-P-positive hepatocytes, which are thought to be precancerous lesions [15]. The hepatic steatosis induced by a high-fat diet was more severe than that induced by a high-fructose diet, but GST-P-positive hepatocytes were more frequent in high-fructose group , suggesting that excess fructose consumption promotes hepatocarcinogenesis to a greater degree.

The number of patients with non-alcoholic, non-viral chronic liver diseases such as NAFLD and NASH has increased with the increase in patients with diseases associated with metabolic syndrome, such as obesity, diabetes, and dyslipidemia. The Japan Public Health Center Study showed that the presence of diabetes increases the risk of HCC, with hazards ratio of 2.24 in males and 1.94 in females compared to those without diabetes [20]. In a comparison of the long-term prognosis in 72 NASH patients and 101 non-NASH patients, Rafiq et al. found no significant difference in overall mortality, but a significantly higher onset of liver-related diseases in the NASH group, with NASH and the presence of type 2 diabetes emerging as independent risk factors for liver disease-related death [21]. Thus, abnormal glucose metabolism seems to be an important risk factor for liver disease, including HCC. In our previous study, an insulin tolerance test showed an increased high-fructose diet-induced insulin resistance [14]; further, fasting blood glucose and insulin levels were significantly higher in the high-fructose group compared to the high-fat group in the current study. These results suggest that hepatocarcinogenesis due to a high-fructose diet involves insulin resistance and/or abnormal glucose metabolism. The association of hepatocarcinogenesis with insulin resistance or abnormal glucose metabolism induced by conditions other than a high-fructose diet requires further investigation.

Previous epidemiological studies have been unable to clarify whether lipid abnormalities are involved in the progression of hepatocarcinogenesis in patients with NASH. In a basic study, Wang et al. compared the number of GST-P-positive hepatocytes in the liver in SD rats given intraperitoneal administration of DEN at 30 mg/kg body weight and then fed with a high-fat or control diet for 6 weeks, in which 71% and 35% of the total calories were derived from fat, respectively [17]. GST-P-positive hepatocytes appeared in both groups, but these cells only formed foci in the high-fat group. Since GST-P-positive hepatocytes are assumed to be precancerous lesions, a high-fat diet may promote development of HCC compared to a control diet. Wang et al. also showed that the increase in foci of GST-P-positive hepatocytes in rats fed a high-fat diet caused an increase in intrahepatic oxidative stress [17]. In our study, more GST-P-positive hepatocytes were present in rats fed a high-fat diet than in controls, but the frequency was higher in those fed a high-fructose diet, in accordance with oxidative stress-induced DNA damage. In contrast, there was a tendency for low expression levels of HO-1 mRNA in the high-fructose group compared to the high-fat group. HO-1 is an oxidative stress marker, but upregulation of HO-1 reduces oxidative stress in organs such as the kidneys in diabetic spontaneously hypertensive rats [22]. Induction of HO-1 also reduces hepatic injury induced by ischemia reperfusion [23] and trauma hemorrhage [24], while inhibition of HO-1 may be associated with acetaminophen-induced liver injury [25]. Furthermore, IFN-γ, which was higher in the high-fructose group compared to the high-fat and control groups in the present study, inhibits both basal and lipopolysaccharide-induced HO-1 expression in macrophages [26]. Therefore, the results of the present study suggest that the possible ability of HO-1 to reduce oxidative stress induced by DEN administration was inhibited by a high-fructose diet. Thus, although high fat and high fructose intake may both induce hepatocarcinogenesis, a high-fructose diet is more likely to be associated with hepatocarcinogenesis through excess oxidative stress.

Three steps of progression from NAFLD to hepatocarcinogenesis have been proposed: initiation, promotion, and progression. Hepatic mRNA expression of IFN-γ tended to be higher in the high-fructose group compared to the other two groups in this study. Intrahepatic IFN-γ mRNA expression has been found to be induced 2 months before the appearance of HCC and IFN-γ may be important in the initiation step of hepatocarcinogenesis in mice exposed to DEN in drinking water [16]. Therefore, high fructose intake may also be associated with this step, rather than promotion and progression. Lipid hyperoxidation and p53 mutation are also associated with initiation [27], but it is unclear whether excess fructose intake is associated with hepatocarcinogenesis through lipid hyperoxidation and gene mutation in our study. Thus, the associations of high fructose intake with all three steps remain to be investigated. In addition, our present and previous [14] studies indicated that hepatocarcinogenesis and delayed liver regeneration were more severe in rats fed a high-fructose diet than in those fed a high-fat diet, although hepatic steatosis was more severe in rats fed a high-fat diet. In contrast, promotion of hepatocyte proliferation through enhanced expression of MAPK-related genes has been associated with hepatocarcinogenesis [17]. Thus, the mechanism or steps of hepatocarcinogenesis may differ between high-fructose and high-fat diets.

Our study has several limitations. First, the frequency of formation of GST-P-positive hepatocyte foci was lower in our model than in that of Wang et al. [17]. This may be due to the difference in route of DEN administration and restriction of food intake. Second, serum triglyceride and fasting blood glucose levels were lower in the high-fat group than those in the control group, in conflict with previous findings [14]. However, Huang et al. also reported that the high-fat diet group had a lower triglyceride concentration than the control group [28]. The total energy comprised of fat and restriction of food intake may influence these results. Third, we used β-actin as a housekeeping gene, but little is known about changes in expression of this gene after DEN or fructose administration. Expression of several genes, including β-actin, has been suggested to increase after DEN administration [29]. However, this change should not affect the significance of our results because all groups in the study received DEN. In addition, GAPDH mRNA initially decreases by almost half and then gradually increases up to 3-fold by 48 h after DEN administration [29]. GAPDH expression may also have differed among the three groups in the present study because GAPDH has roles in activities such as glycolysis and DNA repair that may have differed among the groups [30]. Therefore, β-actin was chosen as a more suitable housekeeping gene compared to GAPDH. Fourth, a high-fructose diet tended to increase IFN-γ mRNA and decrease HO-1 mRNA, but the expression levels were highly variable. IFN-γ protein in liver tissue was also higher in the high-fructose group compared to the high-fat group (data not shown), but the difference was not significant. Further examination of these changes using methods such as immunohistochemistry is required. Finally, it is unclear whether the observed differences in our study are related to an increased incidence of HCC or an increased tumor burden in rats. A longer-term analysis is required to address this issue.

## Conclusions

In conclusion, dietary fructose, rather than dietary fat, enhances hepatocarcinogenesis induced by administration of DEN in rats. This effect seems to be associated with oxidative stress and abnormal glucose metabolism including insulin resistance, rather than the severity of hepatic steatosis. These results suggest that excessive intake of fructose is a potential risk factor for liver cancer in patients with NASH.

## Abbreviations

8-OHdG: Hydroxydeoxyguanosine; ALT: Alanine aminotransferase; DEN: Diethylnitrosamine; GST-P: Glutathione S-transferase placental type; HCC: Hepatocellular carcinoma; HCV: Hepatitis C virus; HO-1: Hemeoxygenase-1; IFN: Interferon; NAFLD: nonalcoholic fatty liver disease; NASH: Nonalcoholic steatohepatitis; SD: Standard deviation; TNF: Tumor necrosis factor.

## Competing interests

HT holds endowed faculty positions in research for HGF tissue repair and regenerative medicine and has received funds from Eisai Co., Ltd.

## Authors’ contributions

RK, HU, KO, RI, ST, SA, SM, KK, MN, TT, and AM carried out the experimental studies. RK, HF, MO, and AI participated in the design of the study and helped to draft the manuscript. HU and HT conceived of the study, oversaw the experimental work, and wrote the manuscript. All authors read and approved the final manuscript.
